# The Impact of Industry Funding on Randomized Controlled Trials of Biologic Therapies

**DOI:** 10.3390/medicines9030018

**Published:** 2022-02-28

**Authors:** Aaron M. Gazendam, David Slawaska-Eng, Nicholas Nucci, Om Bhatt, Michelle Ghert

**Affiliations:** 1 Division of Orthopaedic Surgery, Department of Surgery, McMaster University, Hamilton, ON L8S 4L8, Canada; david.slawaskaeng@medportal.ca (D.S.-E.); ghertm@mcmaster.ca (M.G.); 2 Division of Orthopaedic Surgery, Department of Surgery, University of Toronto, Toronto, ON M5G 1X5, Canada; nwnucci@gmail.com; 3 Faculty of Engineering, McMaster University, Hamilton, ON L8S 4K1, Canada; bhatto1@mcmaster.ca; 4 Faculty of Health Sciences, McMaster University, Hamilton, ON L8S 4K1, Canada

**Keywords:** biologics, immunotherapy, monoclonal antibodies, randomized controlled trials, industry funding, bias

## Abstract

**Background:** There has been substantial interest from the pharmaceutical industry to study and develop new biologic agents. Previous studies outside of the biologics field have demonstrated that industry funding has the potential to impact the design and findings of clinical trials. The objective of this study was to evaluate the impact of industry funding on randomized controlled trials (RCTs) that investigated the efficacy of biologic therapies. **Methods:** A review of all RCTs involving biologic therapies in top impact factor medical journals from January 2018 to December 2020 was performed. The relationship between industry funding and the presence of statistically significant primary outcomes and the use of active comparators were analyzed. **Results:** Among the 157 RCTs included, 120 (76%) were industry funded and 37 (24%) declared no industry funding. Industry-funded studies were significantly more likely to report a statistically significant positive primary outcome compared to studies without industry funding (85% vs. 67%, ***χ*^2^** = 5.867, *p* = 0.015) and were significantly more likely to utilize placebo or no comparator than non-industry-funded trials (78% vs. 49%, ***χ*^2^** = 4.430, *p* = 0.035). **Conclusions:** Industry-funded trials investigating biologic therapies are more likely to yield statistically significant positive outcomes and use placebo comparators when compared to non-industry-funded biologic therapy trials in high-impact medical journals.

## 1. Introduction

Rituximab, an anti-cancer monoclonal antibody, was first approved by the Food and Drug Administration (FDA) for use in the United States in 1997 [1]. Since that time, there has been an exponential growth in the type and number of biologic therapies used [2]. In broad terms, biologics refer to pharmaceuticals in which the active ingredient comes from a living organism [3]. The clinical indications have expanded significantly, with biologic drugs revolutionizing care in oncology, rheumatology, infectious diseases, gastroenterology and respirology. Biologic therapies now encompass a wide variety of pharmaceuticals including monoclonal antibodies, immune cell therapy, vaccines and immune system modulators.

Biologic therapies are expensive interventions and can cost upwards of tens of thousands of dollars per patient, per year [4,5,6,7]. Given the chronic nature of the diseases that biologic therapies are commonly used to treat, long-term treatment is often required. Biologic therapies now make up the majority of total global pharmaceutical sales, with a market value of approximately 1 trillion dollars in 2016 alone, making these therapies one of the most profitable drug classes in medical history [8].

Not surprisingly, there has been significant interest by the pharmaceutical industry in the research and development of new biologic therapies and a significant increase in financial investment in biomedical research [9]. However, there are substantial concerns that industry-funded studies may introduce bias and impact clinical trial results [10]. Evidence from other areas of medical research has demonstrated that industry funding has the potential to impact the design and findings of clinical trials [10,11,12].

The objective of this study was to determine if industry-funded biologic intervention RCTs are more likely to report pro-industry positive results and to use placebo comparators than non-industry-funded RCTs.

## 2. Methods

### 2.1. Study Eligibility Criteria

The inclusion and exclusion criteria for this study were defined a priori. To be included, studies needed to be a randomized controlled trial (RCT) comparing any biologic therapy to any comparator. Biologic agents were defined, as per the National Institutes of Health (NIH), as “A substance that is made from a living organism or its products and is used in the prevention, diagnosis, or treatment of cancer and other diseases. Biologic agents include antibodies, interleukins, and vaccines, also called biological agent and biological drug [13]”. Trials were excluded if they were: (1) nonrandomized controlled trials, (2) non-human trials, or (3) meta-analyses or systemic reviews. No ethics approval was required to perform this study.

### 2.2. Search Strategy

A literature search on RCTs examining biologic pharmaceuticals was performed from 1 January 2018 to 31 December 2020 utilizing PubMed. We chose studies with the greatest potential for scholarly and clinical impact by limiting the search to the three clinical journals with the highest impact factors in 2019 as per the Web of Science, Journal Citation Report: *The New England Journal of Medicine (NEJM), The Lancet,* and *Journal of American Medical Association (JAMA) [14].* Search terms included “biologics”, “anti-TNF”, “vaccine”, “interleukin”, “immunotherapy”, and “monoclonal antibody”. The detailed search strategy is available in Supplementary Materials.

### 2.3. Study Selection

Two independent reviewers (D.S.E, O.B) assessed for study eligibility using the online software Rayyan (2010, Qatar) [15]. Screening was completed in two stages: (1) title/abstract and (2) full text. Discrepancies at the title/abstract stage were resolved by automatic inclusion in the next stage. Discrepancies at the full text stage were resolved through consensus between the reviewers. If this could not be achieved, a senior reviewer (A.G.) was consulted. The inter-reviewer agreement was assessed according to McHugh by calculating a kappa (κ) statistic for the abstract screening stage. The a priori categorization was as follows: κ of 0.81 to 0.99 was considered almost perfect agreement, κ of 0.61 to 0.80 substantial agreement; κ of 0.41 to 0.60, moderate agreement; κ of 0.21 to 0.40, fair agreement; and κ of 0.20 or less, slight agreement [16].

### 2.4. Data Extraction

Data extraction was performed with an online collaborative data extraction form (Google Sheets, Mountain View, CA, USA) that was created and tested prior to extraction [17]. The spreadsheet was designed a priori and piloted by the reviewers with adjustments as needed prior to starting the data abstraction process.

### 2.5. Study Characteristics

Data was extracted on the following study characteristics: area of study (e.g., rheumatology, oncology), sample size, type of biologic therapy, funding sources of the study, and details of the intervention(s) and comparator(s) (placebo vs. other).

A study was deemed industry funded if one of the named sponsors declared by the authors was a manufacturer of one of the trial drugs. All studies without funding or with nonprofit sponsorship (e.g., NIH) were grouped together. The sponsorship declared in the study manuscript was compared to sponsorship details published a priori on https://clinicaltrials.gov (last accessed on 10 December 2021) or other recognized national trial registry databases. The studies were also classified as reporting statistically significant findings for the primary outcome (positive trial), or not reporting statistically significant findings for the primary outcome (negative trial). Outcomes that were not included in the primary analysis were those with a non-inferiority trial design or RCTs designed to assess the safety or tolerability of an experimental drug.

### 2.6. Statistical Analysis

RCT characteristic data is presented using descriptive statistics, with continuous variables reported as a median and range. Any ordinal or nominal variables are represented as median and range. Categorical variables were compared using Pearson’s chi-square tests, and continuous variables were compared utilizing independent samples *t*-tests. Statistical significance was set at *p* < 0.05 for all tests. All statistical analyses were performed utilizing SPSS (IBM SPSS Statistics, Version 23, Armonk, NY, USA).

## 3. Results

The initial search strategy yielded 318 potential studies for inclusion. We identified 157 RCTs published between 1 January 2018 and 31 December 2020 that met the inclusion criteria: 93 from *NEJM*, 54 from *The Lancet*, and 10 from *JAMA* (Table 1). There was almost perfect agreement amongst reviewers for inclusion (k = 0.84, 95% confidence intervals (CI) = 0.80–0.88). The most common areas of study were infectious diseases (29%), oncology (24%), and rheumatology (15%). The majority of studies were international (74%) with patients recruited from multiple countries. The mean sample size of included trials was 1996, range 20–43,548.

### Industry Funding

The authors of 120 (76%) of the included RCTs declared industry funding, whereas 37 (24%) RCTs did not declare any industry funding (Table 2). The mean sample size varied widely but was similar between industry-funded and non-industry-funded trials (2188, range 21–43,548, vs. 1604, range 20–20,019). In all included trials with trial registration information available (n = 154), both industry-funded and those without industry funding, the funding declared by the authors at the time of publication matched the funding information provided at trial registration. One hundred thirty-four studies (91%) were included in the primary outcome assessments. Of the 20 studies not included, 13 were designed to assess safety and 11 were designed to assess non-inferiority. Across all included studies, 110 (82%) had statistically significant primary outcome findings. All statistically significant positive trials favored the intervention group in both industry and non-industry-funded studies. Industry-funded studies were significantly more likely to report a positive primary outcome compared to studies without industry funding (85% vs. 67%, **χ^2^** = 5.867, *p* = 0.015). Subgroup analyses demonstrated no significant differences in the rate of positive primary outcomes between industry funding and no industry funding when evaluating those with an active comparator (85% vs. 71%, *p* = 0.40) or with placebo/no comparator (89% vs. 80%, *p* = 0.20). With respect to the trial comparator, industry-funded studies were significantly more likely to utilize placebo or no comparator than non-industry-funded trials (78% vs. 49%, **χ^2^** = 4.430, *p* = 0.035).

## 4. Discussion

In this study, we reviewed 157 RCTs that assessed the efficacy of biologic therapies in the three highest impact factor medical journals published in the period of 2018–2020. We found that trials with industry funding had higher rates of statistically significant positive findings (all in favor of the biologic intervention) than did trials without industry funding. Industry-funded trials were significantly more likely to utilize placebos or no comparators as the control group.

The presence of industry funding has been associated with positive trial outcomes across a variety of medical fields [11,18,19]. Previous studies have demonstrated no differences in methodologic quality between industry sponsored and non-industry sponsored trials, suggesting that the bias created by industry sponsorship cannot be explained by traditional assessment tools [12,18]. There are several proposed mechanisms by which industry-funded studies have repeatedly been shown to produce positive results. First, selective publication in industry-funded studies continues to be a concern. Industry-funded trials are less likely to publish unfavorable results and often publish favorable results in multiple publications, overestimating the efficacy in subsequent meta-analyses [20,21,22,23,24]. Second, industry-funded studies may use primary outcomes that are more likely to yield a statistically significant outcome but may be less clinically relevant [25]. Many industry-funded trials use surrogate outcome measures as primary outcomes. For example, an industry-funded trial examining the insulin sensitizing drug Rosiglitazone in type 2 diabetics demonstrated a highly statistically significant primary surrogate outcome (fasting plasma glucose). However, secondary outcomes demonstrated no significant clinical benefit over other monotherapies [26].

Perhaps the most prominent and concerning contributor to bias in industry-funded trials is the use of inappropriate comparators. The findings from this review suggest that there continues to be a lack of head-to-head comparative trials in the biologics field [27]. The lack of active comparators in industry-funded trials may play a role in the high rate of favorable results. Utilizing placebo comparators provides advantages to pharmaceutical companies, as they are cheaper, less hazardous, and are more likely to result in favorable results for the biologic arm [10,27]. In addition, evidence gleaned from indirect comparisons across different trials is weaker than direct evidence from head-to-head comparisons, and may be of limited clinical utility when viable alternatives are available to clinicians [28]. Although the subgroup analyses in the current review did not demonstrate differences in industry trials when evaluated by the use of active comparators, our findings were speculative as our analyses were post-hoc and some subgroups contained a small number of observations.

The higher rate of successful positive outcomes in industry-funded trials are not unexpected [25]. Industry funding for clinical trials is provided only for the drugs with the highest likelihood of achieving success. Millions of dollars in research and development and a concerted effort to bring novel therapeutics to market may account for higher levels of “success” when compared to non-industry-funded trials [29]. Given the high cost of pharmaceutical development, involvement of industry is likely a necessity to continue to drive innovation [30].

This review does have a number of limitations. The included trials were limited to the top impact factor medical journals and may not be representative of the literature as a whole. However, given that articles published in high-impact journals are most likely to be further cited, these studies have the greatest potential for clinical impact. A second limitation is the relatively short time period included in this review. Therefore, we were unable to evaluate any possible change over time on the impact of industry funding on published clinical trials in biologic therapy.

## 5. Conclusions

We found that the results of clinical trials in high-impact medical journals that assess the efficacy of biologic therapies are more likely to be positive if the study is industry-funded. Industry-funded trials continue to utilize a high rate of placebo comparators, which may contribute to the high rate of positive trials. These findings should be taken into account when interpreting the evidence surrounding biologic interventions and determining the cost-effectiveness of new biologic therapies.

## Figures and Tables

**Table 1:** Characteristics of included trials.

**Included Trials**		157
**Journal**	*NEJM*	93
	*The Lancet*	54
	*JAMA*	10
**Industry Funding**	*Yes*	120
	*No*	37
**Trial Outcome**	*Positive*	132
	*Negative*	21
**Sample Size (median, range)**		504, 20–43,548
**Country (top 3)**	*International*	116
	*United States*	13
	*China*	6
**Area of Study**	*Infectious Disease*	45
	*Oncology*	38
	*Rheumatology*	28
	*Gastroenterology*	7
	*Respirology*	7
	*Dermatology*	5
	*Other*	35
**Biologic Subtype**	*Monoclonal Antibody*	68
	*Vaccines*	31
	*Small Molecule Inhibitors*	26
	*Enzymes*	9
	*Hormones*	7
	*Fusion Proteins*	4
	*Other*	11

*NEJM = New England Journal of Medicine; JAMA = Journal of the American Medical Association. Positive = trial reported statistically significant primary outcome.*

**Table 2:** Industry-funded vs. non-industry-funded trial characteristics.

		Industry Funding	No Industry Funding	*p*-Value
**Number of Trials**		120	37	
**Journal**	*NEJM*	72	21	
	*The Lancet*	40	14	
	*JAMA*	8	2	
**Trial Outcome**	*Positive*	94	16	0.015 *
	*Negative*	16	8	
	*Not assessed*	7	13	
**Trial Design**	*Superiority*	108	24	
	*Non Inferiority*	4	7	<0.001 **
	*Safety*	7	6	
**Active Comparator?**	*Yes*	37	18	0.035 *
	*No*	79	17	
**Sample Size (mean, range)**		2118, 21–43,548	1604, 20–20,019	0.35

*NEJM = New England Journal of Medicine; JAMA = Journal of the American Medical Association; Positive = trial reported statistically significant primary outcome; Active Comparator = comparator was therapeutically active;* * Statistically significant utilizing Pearson’s chi-squared test. ** Statistically significant utilizing Fisher’s exact test.

## Data Availability

Not applicable.

## Supplementary Materials

The following are available online at https://www.mdpi.com/article/10.3390/medicines9030018/s1, Figure S1: Sample Search Strategy.
