# Supplementary material for: The Impact of Industry Funding on Randomized Controlled Trials of Biologic Therapies
Source: Medicines (Basel). 2022 Feb 28;9(3):18. doi: 10.3390/medicines9030018 (PMC8951352; doi:10.3390/medicines9030018)
Supplement: Supplementary file 1 [file medicines-09-00018-s001.zip › medicines-1605928-supplementary.pdf]

(("biological products"[MeSH Terms] OR ("biological"[All Fields] AND "products"[All Fields]) OR "biological products"[All Fields] OR "biologic"[All Fields] OR "biologicals"[All Fields] OR "biological factors"[MeSH Terms] OR ("biological"[All Fields] AND "factors"[All Fields]) OR "biological factors"[All Fields] OR "biologics"[All Fields] OR "biologically"[All Fields] OR "biology"[MeSH Terms] OR "biology"[All Fields] OR "biological"[All Fields] OR "anti-TNF"[All Fields] OR ("vaccin"[Supplementary Concept] OR "vaccin"[All Fields] OR "vaccination"[MeSH Terms] OR "vaccination"[All Fields] OR "vaccinable"[All Fields] OR "vaccinal"[All Fields] OR "vaccinate"[All Fields] OR "vaccinated"[All Fields] OR "vaccinates"[All Fields] OR "vaccinating"[All Fields] OR "vaccinations"[All Fields] OR "vaccination s"[All Fields] OR "vaccinator"[All Fields] OR "vaccinators"[All Fields] OR "vaccine s"[All Fields] OR "vaccined"[All Fields] OR "vaccines"[MeSH Terms] OR "vaccines"[All Fields] OR "vaccine"[All Fields] OR "vaccins"[All Fields]) OR ("interleukine"[All Fields] OR "interleukines"[All Fields] OR "interleukins"[MeSH Terms] OR "interleukins"[All Fields] OR "interleukin"[All Fields]) OR ("immunotherapy"[MeSH Terms] OR "immunotherapy"[All Fields] OR "immunotherapies"[All Fields] OR "immunotherapy s"[All Fields]) OR ("antibodies, monoclonal"[MeSH Terms] OR ("antibodies"[All Fields] AND "monoclonal"[All Fields]) OR "monoclonal antibodies"[All Fields] OR ("monoclonal"[All Fields] AND "antibody"[All Fields]) OR "monoclonal antibody"[All Fields]) OR ("biological therapy"[MeSH Terms] OR ("biological"[All Fields] AND "therapy"[All Fields]) OR "biological therapy"[All Fields] OR ("biologic"[All Fields] AND "therapy"[All Fields]) OR "biologic therapy"[All Fields])) AND "n engl j med"[Journal]) AND ((randomizedcontrolledtrial[Filter]) AND (2018/1/1:2020/12/31[pdat]))

Figure S1. Sample Search Strategy.
